# Occupational health professionals' attitudes, knowledge, and motivation concerning smoking cessation—Cross‐sectional survey

**DOI:** 10.1002/1348-9585.12145

**Published:** 2020-07-23

**Authors:** Maarit Malin, Nina Jaakkola, Ritva Luukkonen, Antero Heloma, Anne Lamminpää, Kari Reijula

**Affiliations:** ^1^ Department of Public Health University of Helsinki Helsinki Finland; ^2^ The Social Insurance Institution of Finland Helsinki Finland; ^3^ National Institute for Health and Welfare Helsinki Finland

**Keywords:** attitude, knowledge, motivation, occupational health professionals, smoking cessation treatment and support

## Abstract

**Objectives:**

Occupational health (OH) professionals could play a prominent role in smoking cessation treatment and support (SCTS) and help individuals and workplaces become smoke free. However, their role has not been evaluated. The aim of this study was to assess differences between OH professionals' perceptions of their role in SCTS by measuring three groups of OH professionals' attitudes, knowledge, and motivation concerning SCTS.

**Methods:**

We collected data through an online survey completed by a cross‐sectional sample of OH professionals: OH physicians (n = 182), OH nurses (n = 296), and OH physiotherapists (n = 96), collected from national trade union registers. The differences between the OH professional groups were analyzed using ANOVA, the Kruskal‐Wallis, and chi‐square tests.

**Results:**

The OH professionals had a positive attitude toward offering SCTS and were highly motivated to enhance their knowledge of this topic and acquire further training. The OH physicians and OH nurses assessed their current knowledge as sufficient. Conversely, the OH physiotherapists' level of knowledge was seen as insufficient. Traditionally, OH physicians and OH nurses have been responsible for carrying out SCTS, but the majority of the OH physiotherapists thought that SCTS should also be included in their job description.

**Conclusions:**

All the OH professionals were highly motivated to deepen their knowledge of SCTS. The barriers between different professionals need to be recognized in occupational health services (OHS). OHS should organize its SCTS more effectively, strengthen their contributions to smoking cessation programs, and recognize the potential of OH physiotherapists for providing SCTS and enable them to expand their training.

## 1 INTRODUCTION

Tobacco use remains the largest preventable cause of medical disease and cost in developed countries worldwide.^1^, ^2^ Although a significant proportion of smokers want to quit smoking, their attempts to do so are frequently unsuccessful.^3^, ^4^, ^5^, ^6^

Health professionals can potentially play a key role in battling the tobacco epidemic in their everyday health care routines, ^7^, ^8^, ^9^, ^10^, ^11^ as most of them could address tobacco dependence with smoking cessation treatment and support (SCTS) as part of their standard care practice.^7^ The proportion of smokers who receive help from a physician or nurse is too low.^3^, ^12^ When a tobacco user receives advice from two or more health care professionals, the odds of successful cessation are doubled.^14^ Familiarity with standard cessation protocols greatly affects occupational health (OH) professionals' tobacco‐related practices.^15^, ^16^ This requires motivation and opportunity for further training in SCTS, in addition to a positive attitude. A positive attitude and experience of smoking cessation are associated with physicians and nurses actively offering SCTS.^17^, ^18^ However, many barriers exist, such as not regarding SCTS as the health professionals' responsibility ^19^ and lack of time and training.^16^, ^19^ Moreover, smoking cessation interventions are often poorly implemented by health care professionals.^8^, ^13^, ^20^, ^21^

Occupational health services (OHS) are a potentially significant channel for supporting SCTS, for several reasons. First, the primary task of OHS is health promotion and prevention. Hence OH professionals can help more people and do so early in order to prevent serious smoking‐related complications.^22^ Second, OHS have unique opportunities to reach smokers through partnerships with employers to support employees' health. OHS encompass over 90% of the total workforce in Finland. Third, employers have an economic interest in helping their employees quit smoking due to the relationship between smoking and increased sickness absences.^16^, ^23^, ^24^ In addition, personal lifestyle and physical aspects are related to work engagement,^25^ which in turn is associated with work ability.^26^ OHS can both support employees and advise employers when implementing smoking policies and practices.

The revised Tobacco Act of Finland aims to end the use of tobacco and other nicotine products by 2030. In practice, this means that the target is reached if less than 5% of the adult population consume tobacco or nicotine products on a daily basis. Currently, about 14% of the Finnish population smoke daily.^27^ In addition, the Finnish “Tobacco dependence and cessation” 2018 (TDC) Current Care Guidelines ask all physicians to assess tobacco use and dependence, advise cessation to all patients and assess this. This explicitly includes OHS, as they are required by law to play a critical role in increasing work ability. All OH professionals, including OH physicians, OH nurses, and OH physiotherapists, should hence systematically implement SCTS and possess or acquire the knowledge, skills, and correct attitude to help workplaces become smoke free and help smokers quit smoking. This also includes OH physiotherapists, as the contemporary definition of the profession focuses on promoting general health, which includes SCTS.^28^

The study aimed to determine OH professionals' attitudes, knowledge, and motivation concerning SCTS to obtain a deeper understanding of the present state among the key actors in smoking cessation programs. Another aim was to evaluate how these factors vary between different OH professionals (OH physicians, OH nurses, and OH physiotherapists). We sought to increase the efficiency of tobacco use prevention and the counseling services offered by OHS by developing optimized operation models for OHS. The current study investigates how OH professionals see their role in SCTS and what their role could be. The evaluation of current SCTS practices enables the improvement of OH processes and the unfolding of the potential of multiprofessional teams, which could provide a firm foundation for future efforts.

We aimed to answer: Do OH professionals have the knowledge and motivation to provide SCTS? What are their attitudes toward SCTS? We were also interested in any differences in terms of knowledge, motivation, and attitudes among the three OH professions.

## 2 METHODS

A cross‐sectional survey was carried out among OH professionals. OH physicians, nurses, and physiotherapist responded anonymously to an online survey on their knowledge, attitudes, and motivation concerning SCTS.

### 2.1 Participants

Participants were recruited through collaboration with national trade unions for OH professionals, which provided updated registers of active members, including those who were OH physicians, OH nurses, and OH physiotherapists. A member of the national trade union's administrative staff sent an online questionnaire to OH professionals whose e‐mail addresses were available for research projects in the trade unions' membership registers. The respondents' identities were never revealed to the researchers. Administrative staff and respondents who only did administrative work were excluded from the analyses.

The questionnaire was sent to 1290 OH physicians, 1468 OH nurses, and 490 OH physiotherapists. Altogether 182 OH physicians (response rate 15%), 296 OH nurses (21%), and 96 OH physiotherapists (19%) took part in the anonymous, voluntary study.

According to the Finnish Medical Association (FMA), OH physicians are mainly women (67.9%), and in this study, the percentage of female OH physicians was 64.0%. The mean age of OH physicians according to FMA is 52, and in our study, it was 51. According to the Finnish Association of Occupational Health Nurses (FAOHN), the representativeness of women is 99.4%, and in our study, it was 99.0%. The mean age of OH nurses according to FAOHN is 50 and, in our study, it was 48. Correspondingly, the members of the Finnish Association of Occupational Physiotherapists were 89.0% women and in our study, 91.0%, and the mean age of OH physiotherapists was 52.3 and in our study, 52.5.

### 2.2 Questionnaire survey

The questionnaire in the present study was modified from a previously published one.^29^ When, modifying the questions, we also used the studies and material of the Finnish Tobacco Act. The questionnaire was tested together with OH professionals and validated after testing. Attitudes, knowledge, and motivation concerning smoking cessation were subscales of the larger survey. The questionnaire included sections on the OH professionals' smoking cessation practices, such as whether they discussed smoking habits with their patients, and whether time was generally available to discuss this topic. We also included questions on how the Finnish SCTS guideline was currently executed. One section of the wider questionnaire contained questions on the kinds of methods and measuring equipment that are in use. We also asked whether the professionals were aware of the regulations concerning smoke‐free workplaces and whether they collaborated with workplaces to enhance smoke‐free policies. We also examined the collaboration between the OH professionals. These sections of the questionnaire will be reported in another article.

We assessed attitudes to providing additional SCTS in the future using the following statements: “In my work I am committed to promoting smoke‐free practices at the workplace”, “I would like to emphasize smoke‐free practices in several OHS processes”, and “I am likely to emphasize that not smoking enhances well‐being at work and that SCTS is a part of every patient's medical treatment plan” (Table 2) We used a Likert scale: 1 = completely disagree, 2 = somewhat disagree, 3 = neither agree nor disagree, 4 = somewhat agree, or 5 = completely agree.

We also asked the OH physiotherapists whether SCTS was included in their current job description and whether they thought it should be included. We elicited the respondents' demographics, total OHS work experience, and smoking habits (Table 1).

**Table 1:** OH professionals' background information

	All	OH Physicians	OH Nurses	OH Physiotherapists	*P*‐value
	(n = 574)	(n = 182)	(n = 296)	(n = 96)	
Gender (male/female), %	13/87	36/64	1/99	9/91	<.001*
Age, years Mean ± SD	49.7 ± 9.2	51.0 ± 9.1	48.0 ± 9.6	52.5 ± 6.8	<.001**
Working years Mean ± SD	15.3 ± 9.7	15.8 ± 10.0	14.3 ± 9.2	16.4 ± 10.0	.089**
Smoking (currently/ ex‐smoker/never), %	5/9/86	4/7/90	6/12/82	1/6/93	.116*

* *P*‐values are for the chi‐square test.

** *P*‐values are for the F test.

**Figure 1:**
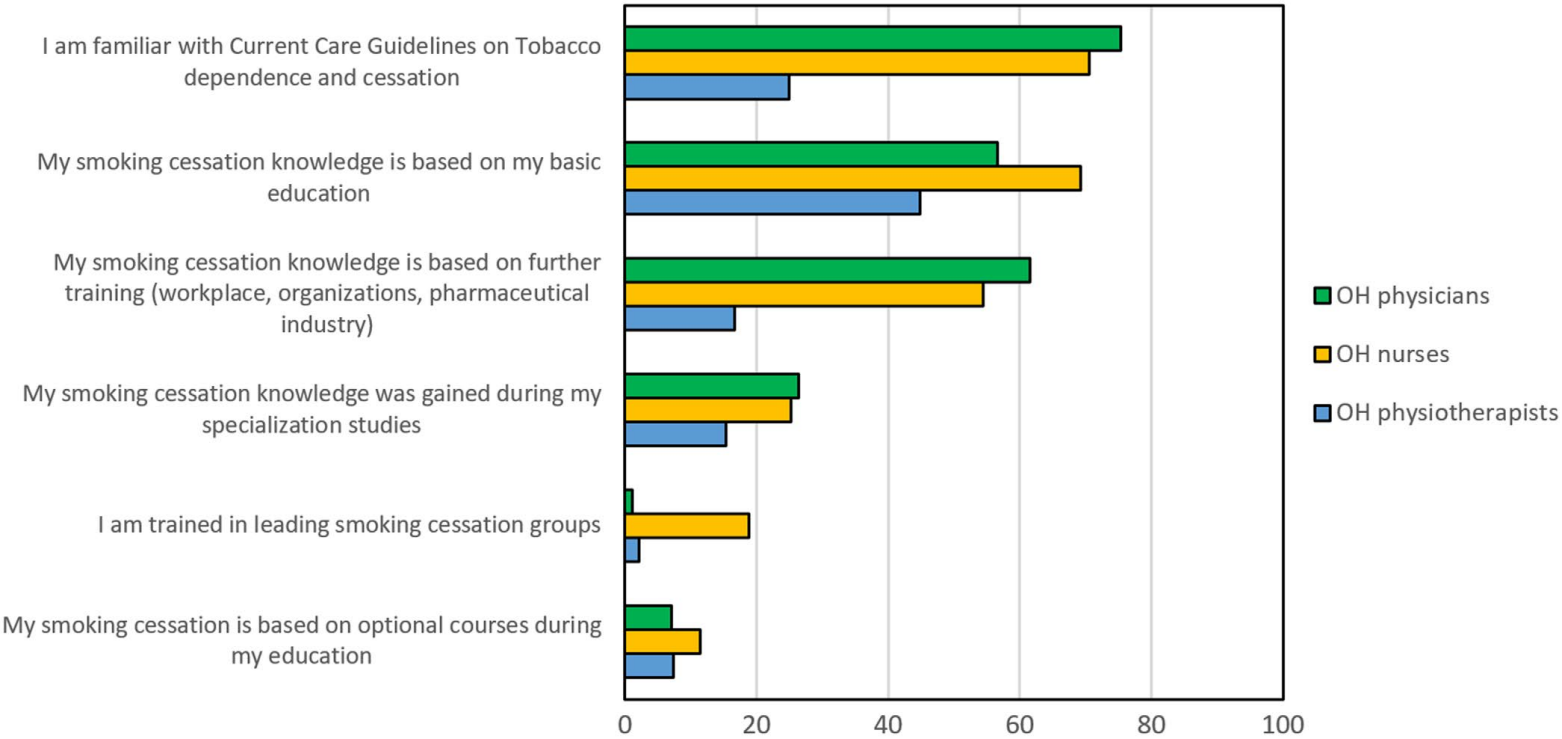
OH professionals' knowledge regarding SCTS

Knowledge of SCTS was assessed by asking about the professionals' familiarity with the Finnish Current Care Guidelines on Tobacco^30^ and what their knowledge was predominantly based on: basic education, further training (workplace, organizations such as trade unions, pharmaceutical industry), or optional courses during education or during specialization studies. We also asked if the respondents had trained in leading smoking cessation groups (Figure 1).

The response options were “yes” or “no”.

**Figure 2:**
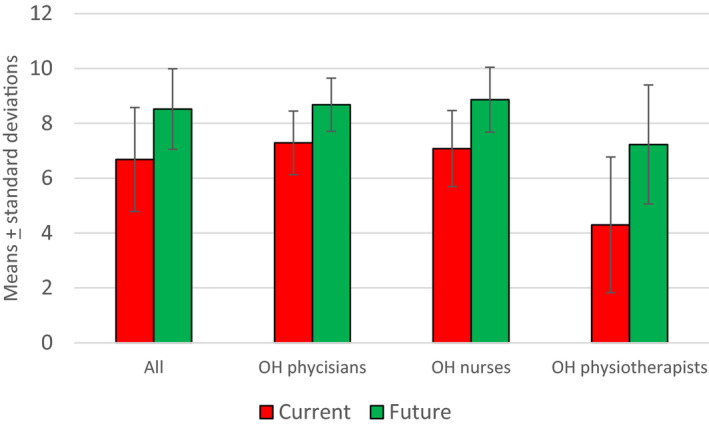
Perceived current and targeted future knowledge among OH professionals

The assessment scale for current and target levels of knowledge was from 0 to 10 (Figure 2). The questions were as follows: “How do you assess your current knowledge?” and “At what level would you like your future knowledge to be?”

We asked about the participants' motivation to receive further training in several areas using statements (Table 3). We asked whether they were motivated to learn more about one or more of these subjects: the theoretical background of SCTS, smoking cessation counseling methods (motivational interview), measuring equipment, providing information on the availability of SCTS at the workplace, assessing the general effectiveness of SCTS among patients/at workplaces, and training that provides expertise in smoking cessation. We used the following Likert scale to evaluate motivation for further training: 1 = completely disagree, 2 = somewhat disagree, 3 = neither agree nor disagree, 4 = somewhat agree, or 5 = completely agree.

### 2.3 Procedure

The present study was conducted in two phases, in 2013 and in 2017. In 2013, the OH physicians and OH nurses received an invitation to answer an online questionnaire on SCTS. In 2017, the same invitation was sent to OH physiotherapists. The inclusion of OH physiotherapists was fundamental, as an OHS team primarily consists of these three health care professionals. The Ethics Committee of the University of Helsinki in the Faculty of Medicine approved the research plan.

### 2.4 Statistical analysis

Descriptive data are presented as mean ± standard deviations in continuous variables, and the percentages in categorical variables. To analyze the differences between the three OH professional groups, we performed one‐way analysis of variance (ANOVA) for normally distributed variables, the Kruskal‐Wallis test for non‐normal continuous variables, and the chi‐square test for categorical variables.

Our questionnaire included several possible subscales. This study used explanatory factor analysis to determine possible attitude and motivation factors (since our questionnaire included many possible variables). When performing explanatory factor analysis of the 16 statements, we determined the attitude and motivation subscales. The attitude subscale consisted of four statements and the motivation subscale of six statements. Cronbach's alpha (internal consistency coefficient) was used as a measure of subscale reliability. The knowledge subscale was continuous scale, and we performed Mann‐Whitney tests when comparing perceived current and targeted future knowledge among OH professionals.

When handling the statements on motivation and attitude, we first evaluated each statement separately. Next, we combined the motivation and attitude statements into two mean scores. The new values varied from 1 to 5. The mean value of the motivation statements consisted of the six statements mentioned above. Cronbach's alpha was 0.85. The mean value of attitude toward SCTS consisted of four statements and Cronbach's alpha was 0.756. With these combined scores, we used the Mann‐Whitney U test to determine any statistically significant differences between OH physicians and OH nurses or between OH physicians and OH physiotherapists.

A *P*‐value of < .005 was considered statistically significant. We conducted all the analyses using SPSS (version 25) software (IBM Corporation, New York).

## 3 RESULTS

### 3.1 Respondents' background information

Most of the OH physicians were women (64%) and their mean age was 51. The OH nurses were mostly women (99%) and their mean age was 48. The OH physiotherapists were also mostly women (91%) and their mean age was 52.5. The OH nurses were younger than the OH physicians and OH physiotherapists, but their working years in different occupations hardly deviated from each other. The current smoking prevalence among the OH physicians was 0.5% and 1% among the OH nurses. None of the OH physiotherapists reported being current smokers. There were more occasional smokers among the OH nurses (5.1%) than the OH physicians (2.7%) and OH physiotherapists (1.0%). The majority of the OH professionals were women.

The demographics of the professionals who answered the questionnaire were consistent with those of the professionals who were trade union members.

### 3.2 Attitudes toward SCTS

The scores for all the attitude variables were high and we detected only slight differences between the OH professionals. Standard deviations were minor and the answers mainly had a value of 4 or 5.

The attitudes toward nonsmoking and well‐being at work of the OH physicians differed from those of the OH physiotherapists (Table 2). The number of nurses was higher than the number of OH physicians and OH physiotherapists and due to the fact that the slight differences were statistically significant.

**Table 2:** Mean values ± standard deviations of attitude variables among OH professionals

	All	OH physicians	OH nurses	OH physiotherapists	*P*‐value
	(n = 574)	(n = 182)	(n = 296)	(n = 96)	
Mean value of attitude variables	4.7 ± 0.5	4.7 ± 0.5	4.7 ± 0.5	4.6 ± 0.5	.273
Attitude variables					
No smoking and well‐being at work	4.8 ± 0.5	4.7 ± 0.6	4.8 ± 0.4	4.8 ± 0.6	.005
Smoke‐free practices at workplaces	4.7 ± 0.7	4.7 ± 0.6	4.7 ± 0.6	4.6 ± 0.9	.299
Smoke‐free practices at OHS processes	4.7 ± 0.6	4.6 ± 0.6	4.7 ± 0.5	4.5 ± 0.9	.160
SC and patients' medical treatment plan	4.6 ± 0.7	4.7 ± 0.5	4.6 ± 0.7	4.4 ± 0.7	.300

Note

*P*‐values are for the Kruskall‐Wallis test.

Overall, 93.8% of the respondents reported that SCTS should be included in their job description.

However, 42.7% of the OH physiotherapists responded that SCTS was not currently included in their job description.

### 3.3 Current and target level of knowledge regarding SCTS

The OH physicians and nurses assessed their familiarity with SCTS information based on the current Finnish TDC guidelines as sufficient. A greater number of OH physicians reported that their knowledge was based on further training, for example, at their workplaces, in organizations such as trade unions, or in the pharmaceutical industry. The OH physiotherapists had participated in further training to a lesser degree than the OH physicians and OH nurses. Training and education during specialization studies was low among all the groups. Figure 1 shows how the OH professionals assessed their knowledge of SCTS information, which is expressed as a percentage. The figure describes what the knowledge was based on and the level of familiarity with Current Care guidelines.

All the participants assessed their current knowledge regarding SCTS and the level at which they would like it to be in the future. The OH physicians' self‐assessment of current knowledge was the highest (mean 7.3, SD = 1.2), followed by that of the OH nurses (mean 7.1, SD = 1.4) and the OH physiotherapists (mean = 4.3, SD = 2.5). The participants were highly motivated to enhance their skills. The OH physiotherapists wanted to increase their mean value to 7.2 (SD = 2.2) (Figure 2). The OH physicians were motivated to increase their smoking cessation skills to 8.7 (SD = 1.0) and the OH nurses to 8.9 (SD = 1.2). The OH physiotherapists' self‐assessed level of current knowledge was the lowest, but at the same time, they were motivated to increase their knowledge closer to the level of the OH physicians and OH nurses.

Figure 2 shows how the OH physicians and OH nurses assessed their current knowledge as sufficient, but were still motivated to obtain further knowledge. The OH physiotherapists' estimation of their current knowledge differed from that of the other two groups. This result is statistically significant (Mann‐Whitney test, *P* < .0001). All the groups were highly motivated to enhance their current knowledge.

### 3.4 Motivation in specific topics

Table 3 shows the level of the OH professionals' motivation to receive further training. The OH nurses were particularly motivated to receive further SCTS training. Their mean value of motivation variables was the highest, at 3.9 (SD = 0.8). The differences between the mean values of the motivation variables of the OH physicians and OH nurses were statistically significant (*P* = .001), but those between the OH physicians and OH physiotherapists were not (*P* = .218).

We also examined single variables for motivation to determine the training topics in which the OH professionals were most likely to participate. Among all three groups of OH professionals, motivation was the highest for methods (motivational interview), effectiveness, and evaluation. The lowest scores were in obtaining expertise in smoking cessation.

**Table 3:** Mean values ± standard deviations of motivation variables among OH professionals

	All	OH physicians	OH nurses	OH Physiotherapists	*P*‐value
	(n = 574)	(n = 182)	(n = 296)	(n = 96)	
Mean value of motivation variables	3.7 ± 0.9	3.6 ± 0.9	3.9 ± 0.8	3.4 ± 1.1	<.001
Motivation variables					
Methods (motivational interview)	4.2 ± 1.0	4.1 ± 1.0	4.4 ± 0.9	3.8 ± 1.2	<.001
Effectiveness and evaluation	4.0 ± 1.0	4.0 ± 1.0	4.1 ± 0.9	3.6 ± 1.3	.001
Theoretical background	3.7 ± 1.1	3.5 ± 1.2	3.8 ± 1.1	3.6 ± 1.1	.029
Measuring equipment	3.7 ± 1.2	3.6 ± 1.1	3.9 ± 1.1	3.4 ± 1.3	<.001
Marketing to client's company	3.5 ± 1.2	3.5 ± 1.3	3.7 ± 1.1	3.3 ± 1.3	.036
Obtaining expertise	3.3 ± 1.3	3.1 ± 1.4	3.6 ± 1.2	3.0 ± 1.3	<.001

Note

*P*‐values are for the Kruskal‐Wallis test.

The motivation variables describe the participants' motivation to receive further training in several topics.

## 4 DISCUSSION

To the best of our knowledge, this is the first study to assess and compare the factors associated with SCTS among OH professionals, including OH physiotherapists. The main findings are as follows. Firstly, for the OH physicians and OH nurses, SCTS is part of their job description; they have a positive attitude toward contributing to smoking cessation and are highly motivated to enhance their knowledge of and skills in this topic. Secondly, the OH physiotherapists reported being almost as motivated as their OH physician and OH nurse colleagues, but rated their knowledge and skills as significantly lower. Both conclusions show potential for further increasing the role of OH professionals in SCTS. We next discuss each of these conclusions in detail.

Most OH nurses believed they play a key role in carrying out SCTS, which is in line with earlier studies.^16^ Educational programs focus on the OH nurses' role in supporting smoking cessation efforts, and nurses engage in interventions to help smokers quit.^31^ The OH physicians saw SCTS as a natural part of their job and they assessed their knowledge as sufficient. These findings are in line with other research indicating that primary care physicians are more active in SCTS than those who work in secondary health care.^17^

The attitudes of the OH professionals toward SCTS were very positive, and generally, their smoking prevalence was very low. This is consistent with other studies that have shown that smoking habits among health care professionals correlate strongly with their attitudes toward providing SCTS practices.^32^, ^33^, ^34^, ^35^ We found that the scores for all the attitude variables were high and that the differences between the OH professionals were only small. The OH professionals had a positive attitude toward implementing more SCTS than they currently provided. Interestingly, standard deviations were low among all the OH professional groups.

Although the Finnish TDC guideline is available to all OH professionals, the OH physicians and OH nurses were the most familiar with them. Accordingly, the OH physicians' and OH nurses' self‐assessed knowledge of the Finnish TDC guideline was excellent, and their motivation to learn and do more in terms of SCTS was particularly high. The OH physiotherapists' familiarity with the TDC guideline was insufficient, which is consistent with other studies.^19^ Training and familiarity with standard cessation protocols significantly affects health care professionals' tobacco‐related practices.^6^, ^8^, ^15^, ^16^, ^36^ The responses of the OH physicians and OH nurses regarding their familiarity with the Finnish TDC guideline were positive, which may be due to their positive attitudes to SCTS in general. This presents an opportunity to develop training programs on this topic.

The knowledge of OH nurses and physiotherapists was mostly based on basic education, which may mean it is outdated and might present implementation challenges in their daily practices. The OH physiotherapists had not participated as much in further training, and thus had fewer opportunities to obtain sufficient knowledge of SCTS. Health professionals engage in interventions to help smokers quit by organizing training programs.^31^, ^37^ In practice, all OH professionals should have the same opportunities for further training related to SCTS. An interesting finding was that gaining knowledge during OH specialization studies was very low among all groups. When implementing creditable SCTS, OH professionals require adequate knowledge besides the positive attitude and motivation.

All the OH professional groups were motivated to enhance their knowledge of SCTS. These results are consistent with previous studies which have identified individual skills and motivation as the most important predictors of implementation.^18^ Using explanatory factor analysis, we determined the domains about which the OH professionals would like to learn more: methods, evaluating effectiveness, theoretical background, measuring instruments, marketing to client companies, or obtaining expertise. Regarding further training, all the OH professionals were particularly motivated to improve their skills in motivational interviewing. They were also motivated to enhance their skills in evaluating SCTS effectiveness. The OH physiotherapists were also interested in further training in the correlations between smoking and spinal disease. The need for further training may indicate a sense of inadequacy regarding the OH professionals' skills when discussing SCTS with their patients.

OH professionals can significantly impact the health of their clients by using evidence‐based smoking cessation interventions. Even though legislation is in place, the number of OH professionals who provide SCTS could be higher in practice. Problems with implementation might be related to individual variance among the OH professionals' lack of knowledge and training, or organizational barriers such as the absence of protocols.^8^, ^18^, ^38^ OHS and workplaces already collaborate in development programs for enhancing well‐being at work, stress management, and employees' behavior change interventions, which are executed multiprofessionally. A multiprofessional approach to tobacco cessation programs should involve an OH physician, an OH nurse, and an OH physiotherapist. Working together as a team at workplaces would enhance cessation counseling guidelines and improve the health and welfare of both individuals and the community.^39^ Because OH professionals work in multiprofessional teams in a clinical setting, training and educating all health professionals within multiprofessional models would be prudent.^40^ Our main message is that OH professionals have great potential for providing SCTS. However, the level of knowledge of OH professionals varies greatly, particularly among OH physiotherapists. On the other hand, the OH physiotherapists wanted to increase their knowledge regarding SCTS and wanted to share their expertise and take part in multiprofessional SCTS in OHS in collaboration with OH physicians and OH nurses.

### 4.1 Strengths and limitations

This seems to be the first study to assess the attitudes, knowledge, and motivation concerning SCTS of OH professionals, including OH physiotherapists. The survey covered a wide spectrum of OH professionals in Finland, addressing the demographic data of the respondents, and dividing OH professionals into OH physicians, OH nurses, and OH physiotherapists. Furthermore, the respondents' smoking status corresponded to the national state level.

Electronic surveys have limitations such as generally lower response rates, and this was also a limitation in the present study. It may also be possible that the OH physicians' and OH nurses' knowledge of SCTS was so good because these respondents were more interested in SCTS in general. It is possible that the participants who enrolled for this study might differ from average OH professionals in meaningful ways. The fact that the participants were self‐selected may limit the generalizability of the findings. Although the response rate was low at under 20%, the data were consistent with professionals who are members of trade unions. Despite the limitations of this study, the results may contribute to more effective approaches to training OH professionals in SCTS.

## 5 CONCLUSIONS

To conclude, all the OH professionals had a positive attitude toward STCS. The OH physicians and OH nurses assessed their current knowledge as sufficient but were still motivated to undergo further training and enhance their knowledge regarding smoking cessation. The OH physiotherapists perceived their current knowledge as insufficient but were motivated to increase their knowledge to the level of OH physicians and OH nurses. However, the OH physiotherapists seemed to be an unused resource in SCTS. This research validates the need to implement SCTS, including further training and continuing multiprofessional development and education, in order to support the delivery of smoking cessation interventions by OH professionals at the workplace. Further research should examine effective strategies for implementing organized multiprofessional models in OHS and health care. Indeed, all health care professionals are needed to encourage smokers to quit smoking.

## DISCLOSURE

*Ethical approval:* All the procedures carried out in the study that involved human participants were in accordance with the ethical standards of the institutional research committee and with the 1964 Helsinki declaration and its later amendments or comparable ethical standards. *Informed consent:* Written informed consent was obtained from all the participants with full disclosure and explanation of the purpose and procedures of this study. We explained that their participation was voluntary, and that they could withdraw from the study at any time, even though participation was voluntary without any reason. *Registry and the Registration No. of the study/Trial:* N/A. *Animal Studies:* N/A. *Conflict of Interest:* The authors declare that they have no competing interests regarding this study. *Funding:* The author(s) received no financial support for the research, authorship, and/or publication of this article.

## AUTHOR CONTRIBUTIONS

K.R, AH, and MM conceived the ideas; MM and NJ collected the data; MM, RL, and AL analyzed the data; MM, RL, AL, and KR led the writing.
